# Uniformity Masks Design Method Based on the Shadow Matrix for Coating Materials with Different Condensation Characteristics

**DOI:** 10.1155/2013/160792

**Published:** 2013-10-08

**Authors:** Lichao Zhang, Xikun Cai

**Affiliations:** State Key Laboratory of Applied Optics, Changchun Institute of Optics, Fine Mechanics and Physics, China Academy of Sciences, Changchun 130033, China

## Abstract

An intuitionistic method is proposed to design shadow masks to achieve thickness profile control for evaporation coating processes. The proposed method is based on the concept of the shadow matrix, which is a matrix that contains coefficients that build quantitive relations between shape parameters of masks and shadow quantities of substrate directly. By using the shadow matrix, shape parameters of shadow masks could be derived simply by solving a matrix equation. Verification experiments were performed on a special case where coating materials have different condensation characteristics. By using the designed mask pair with complementary shapes, thickness uniformities of better than 98% are demonstrated for MgF_2_ (*m* = 1) and LaF_3_ (*m* = 0.5) simultaneously on a 280 mm diameter spherical substrate with the radius curvature of 200 mm.

## 1. Introduction

Thickness uniformity control of optical coatings is of great importance for precision optical applications [1, 2]. Many works have been done to enhance coating uniformities for the evaporation coating technique. Most of these works were focused on two directions. On one hand, simple rotation [3] or planetary rotation [4] of substrate fixtures was used to get optimized thickness distributions. On the other hand, sufficient uniformities may not be achieved by merely rotating substrates in some cases, especially when substrates are large or strongly curved. Shadow masks were often used in these cases [5–9]. Up to now, the design of shadow masks is a challenging work yet. There are still two problems that need to be solved. For one thing, the design procedure is usually a laborious process because the shadow effects of masks on substrates are inexplicit. This makes the mask design an inconvenient process that usually involves complex mathematic algorithms, such as the analytical method developed by Villa et al. [6] for flat and curved substrates with simple rotation movement and the iterative approach adopted by Bauer et al. [7] or the initialize-and-mending design procedure proposed by both Liu et al. [8] and Guo et al. [9] for curved substrates with planetary rotation motion. For another thing, mask design methods used in previous works are not effective in the cases where coating materials used in the same deposition process have different values of condensation parameters, because the model popular used to describe the deposition rate in these methods is a simplified model which does not take the condensation characteristics [10] of coating materials into consideration.

We develop an approach to solve the two problems. By appropriate partitioning of the substrate and the mask, a shadow matrix could be introduced. The shadow matrix contains coefficients that build quantitive relations between shape parameters of mask partitions and shadow quantities of substrate partitions one by one. By using this method, a mask pair with complementary shapes could be designed for specified surface figures to get the control of uniformities for two materials with different condensation parameters. Verification experiments of the proposed method were made on a convex spherical substrate with the radius curvature of 200 mm by using the planetary rotation fixture. Thickness uniformities of better than 98% were achieved for both MgF_2_ (*m* = 1) and LaF_3_ (*m* = 0.5) on the diameter of 280 mm. In this paper, we describe the design procedure of masks and present thickness uniformity measurement results.

## 2. Theory and Experiment

The design of shadow masks begins with the deposition rate that is expressed as [10]

(1) $$\begin{array}{c} t_{p}=K\frac{\cos^{n}{\alpha \cos}^{m}\beta }{r_{p}^{2}}, \end{array}$$

where *t*_*p*_ is the film thickness at the point *p*, *K* is a constant, *α* and *β* are the emission and condensation angle of coating particles to normals of the source and the substrate, respectively, *n* and *m* describe the emission and the condensation characteristics, respectively, and *r*_*p*_ is the distance from point *p* to the evaporation source. While the emission characteristics parameter *n* is well recognized, the condensation characteristics parameter *m* is usually ignored in the literatures; that is, it is usually assumed to be 1 as default. In fact, although the value of the condensation parameter equals 1 in most cases [7], other *m* values that are in the range of 0.5~1 [10] could be found under some deposition processes occasionally. 

**Figure 1:**
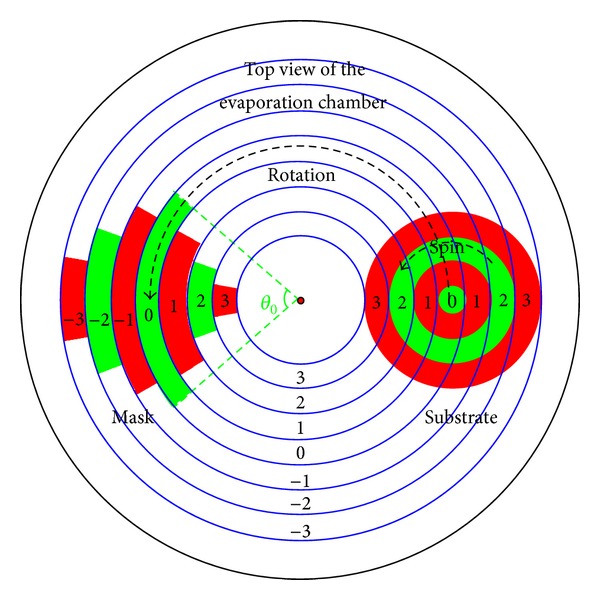
Partition arrangement of the mask and the substrate.

The most challenging problem on the design of shadow masks is how to establish quantitative relation between portions shadowed on each position of the substrate and shape parameters of the mask. Here we propose a shadow matrix to solve the problem. The shadow matrix is a matrix that corresponds to a given partition arrangement that divides the substrate and the mask into several partitions, respectively, as illustrated by the example in Figure 1.

In this partition arrangement, the substrate could be divided into *k* pieces of concentric cirques. In the planetary rotation geometry, projections of these cirques will sweep on the mask plane to form a set of trajectories, which have shapes just like tracks for athletic running. It is a natural idea to divide the mask into 2*k* − 1 pieces according to these tracks. If they are numbered as −*k* to *k*, we could find that two pieces with numbers having the same absolute value are correspondent to the same substrate partition. For example, mask partition of number −*k* and number *k* corresponds to the same substrate partition of number *k*. Therefore, mask partitions could be combined and renumbered from 1 to *k*, so that they could correspond to *k* pieces of substrate partitions one by one. Then the shadow equation could be written as

(2) [S](θ)=[S11S21⋯Sk1S12⋯⋯Sk2⋯⋯⋯⋯S1k⋯⋯Skk](θ1θ2⋯θk)=(Tθ1_to_t1+Tθ2_to_t1+⋯Tθk_to_t1Tθ1_to_t2+Tθ2_to_t2+⋯Tθk_to_t2⋯Tθ1_to_tk+Tθ2_to_tk+⋯Tθk_to_tk)=(T1T2⋯Tk)=(T),

where [*S*], (*θ*), and (*T*) are the shadow matrix, shape parameter vector of the mask, and the shadow portion vector, respectively. Each element *θ*_*i*_ in (*θ*) is the shape angle of the *i*th mask partition, as shown in Figure 1. Each element *T*_*j*_ in (*T*) is the variation of the shadowed portion on the *j*th substrate partition caused by the overall shadow effect of the whole mask. The value of *T*_*j*_ is the sum of *k* terms, each one term is the product of an element in [*S*] and an element in (*θ*). For example, *T*_*θi*_to_*tj*_ = *S*_*ij*_ × *θ*_*i*_. Each element *S*_*ij*_ in [*S*] is the shadow coefficient of the *i*th mask partition to the *j*th substrate partition. This shadow coefficient is the scale factor that equals the variation of *T*_*j*_ on the *j*th substrate partition, when the value *θ*_*i*_ of the *i*th mask partition is added or substracted by a unit degree.

According the definitions above, the shape of the shadow mask could be derived easily by the following routine: first, calculate the normalized thickness distribution vector (*T*_original_), and then the shadow portion vector (*T*) could be obtained by substracting (*T*_original_) on the desired thickness distribution (*T*_target_); second, calculate values of each element in [*S*] by calculating values of *T*_*j*_ under some different values of *θ*_*i*_ and liner fitting the relation of *θ*_*i*_ versus *T*_*j*_, and then *S*_*ij*_ equals the slope of the fitting result; last, derive mask shape parameters by solving (2) reversely; that is,

(3) $$\begin{array}{c} \left(\theta \right)={\left[S\right]}^{-1}\left(T\right). \end{array}$$

Then the mask shape parameter vector (*θ*) could be derived by solving (3) directly.

In case two materials (high and low refractive index materials) used in the coating process have different *m* values, (*T*_original_) vectors of two materials are different for the same surface figure. This leads to the differences of [*S*] and [*T*] in (2) for the two materials. Obviously, no numerical solution of could satisfy the relation of (2) for both materials at the same time. Therefore, two masks with complementary shapes are needed to achieve uniformities control for both materials simultaneously. Then the shadow equation should be rewritten to a shadow equation set as

(4) $$\begin{array}{c} \left[S_{M1\_\text{to}\_H}\right]\left(\theta_{M_{1}}\right)+\left[S_{M2\_\text{to}\_H}\right]\left(\theta_{M_{2}}\right)=\left(T_{H}\right)\\ \left[S_{M1\_\text{to}\_L}\right]\left(\theta_{M_{1}}\right)+\left[S_{M2\_\text{to}\_L}\right]\left(\theta_{M_{2}}\right)=\left(T_{L}\right), \end{array}$$

where *M*1 and *M*2 represent two different masks and (*θ*_*M*1_) and (*θ*_*M*2_) are their shape parameters, respectively. (*T*_*H*_) and (*T*_*L*_) are shadow portion vectors for high and low refractive index materials, respectively. Four shadow matrices used in the equation set are shadow matrix of two masks for two materials, respectively.

Therefore, shape parameters could be derived easily by solving equation set (4); that is,

(5) (θM1)=([SM1_to_H]−[SM1_to_L])−1×{[SM2_to_H]−1(TH)−[SM2_to_L]−1(TL)},(θM2)=([SM2_to_H]−[SM2_to_L])−1×{[SM1_to_H]−1(TH)−[SM1_to_L]−1(TL)}.

The shadow mask design and verification procedure were performed by a coating plant (SYRUSpro 1110 DUV by Leybold Optics). The 1100 mm diameter evaporation chamber contains a planetary rotation fixture carrying four substrate holders, and the distance between each spin rotation axis and the revolution axis is 300 mm. MgF_2_ and LaF_3_ coatings were deposited by molybdenum boats onto 25 mm diameter DUV grade fused silica substrates. Substrates were distributed along the axis on a convex spherical jig with the radius curvature of 200 mm. To determine thickness distributions, single layer MgF_2_ and LaF_3_ coatings were deposited onto substrates. The vertical distance between evaporation sources and the substrate holder is 700 mm. Planes that contain shadow masks are 10 mm below the lowest point of the spherical jig. Transmissions of coatings were measured with spectrophotometer (Lambda 1050 by Perkin Elmer) and layer thickness was derived by fitting transmission spectrum with the commercial thin film software Optilayer.

We wrote a computation code to calculate coating thickness distributions and shadow matrix in (2) or (4). In the code, geometry arrangement parameters of the coating chamber and evaporation sources, together with values of *n* and *m*, are taken into account. The code is introduced in detail elsewhere [11].

## 3. Results and discussion

**Figure 2:**
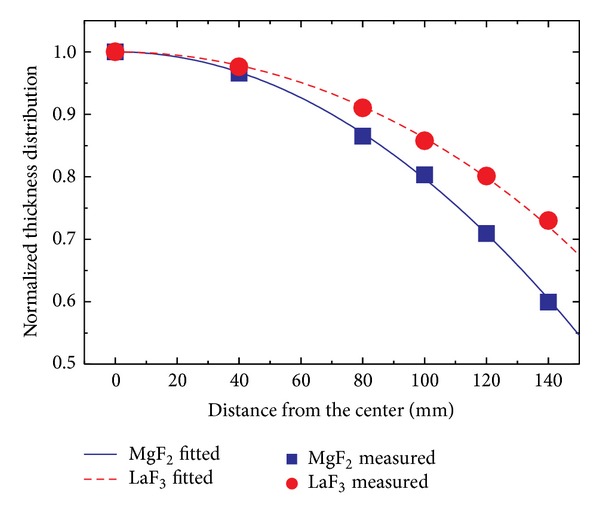
Measured and fitted results of normalized thickness distributions for MgF_2_ and LaF_3_ coatings.

Determination of emission parameters and condensation parameters is the perquisition to the design of shadow masks. These parameters are acquired by fitting thickness distributions without shadow masks, as shown in Figure 2.

In our experiment, values of emission parameters and condensation parameters were determined to be *n* = 2 and *m* = 1 for MgF_2_ and *n* = 2 and *m* = 0.5 for LaF_3_ by fitting thickness distributions without shadow masks.

**Figure 3:**
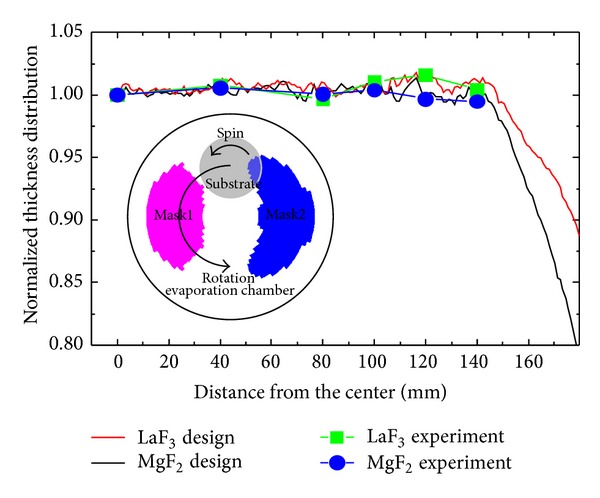
Designed and measured uniformity distributions of MgF_2_ and LaF_3_ single layers, together with shadow masks shown in the inserted figure.

By using these parameters, shadow matrix and vectors needed to derive shape parameters in (5) were calculated. In practices, the 280 mm convex spherical substrate and two shadow masks were divided into seven partitions. Shapes of designed mask pair by using (5), together with designed and measured thickness distributions with the correction of masks, are shown in Figure 3.

**Figure 4:**
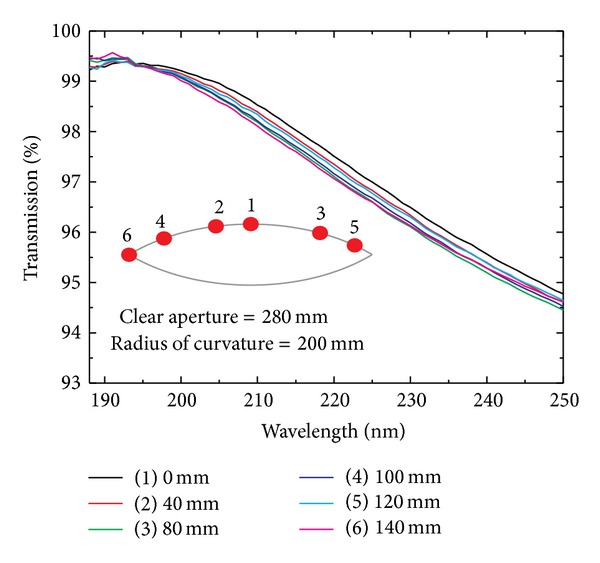
Transmissions of both side AR coated samples on a convex surface, measured at positions shown in the inset.

By using the mask pair with complementary shapes, thickness distributions of both MgF_2_ and LaF_3_ were well tuned simultaneously. Measured thickness uniformities were better than 98%, which were in good agreement with the designed ones. To verify optical performances under this thickness control, additional test was performed by designing and depositing antireflection (AR) coatings. Transmission spectrum of both side AR coated samples are illustrated in Figure 4.

The central wavelength of AR coatings for transmission spectrum is in good agreement with the designed wavelength of 193 nm, and transmission difference measured at 193 nm of all samples is less than 0.1%. These results show that uniform coating thickness yields uniform optical performances. Meanwhile, there are still some differences among these samples, when comparing their overall transmission spectrum on the wavelength region far from the design wavelength. This is due to their refractive index difference caused by the difference of incidence angle range of coating particles along the substrate axis [12], which is usually observed for coatings on strongly curved surface [8].

## 4. Conclusion

We have proposed an approach method to design shadow masks based on the concept of the shadow matrix. By solving an equation set that contains four shadow matrix, thickness distributions adjustment for two coating materials with different condensation parameters could be achieved simultaneously. By using this method, the design of shadow masks becomes a rather simple and straightforward routine. We have applied this method to a convex spherical substrate with the diameter of 280 mm and the radius curvature of 200 mm with the coating material combination of MgF_2_ (*m* = 1) and LaF_3_ (*m* = 0.5). Well-designed masks with complementary shapes yielded a standard of better than 98% on thickness uniformity control for both coating materials simultaneously. The proposed method is a general approach that could be applied to control thickness uniformities of any surfaces that have rotary symmetrical shapes, by using either simply rotation or planetary rotation deposition systems. The method could be easily extended to curved surfaces with other shapes to meet thickness uniformity requirements in precision optical applications.
